# Subtyping of Parkinson’s Disease - Where Are We Up To?

**DOI:** 10.14336/AD.2019.0112

**Published:** 2019-10-01

**Authors:** Elizabeth Qian, Yue Huang

**Affiliations:** ^1^School of Medical Science, Faculty of Medicine, UNSW Sydney, 2032, Australia.; ^2^China National Clinical Research Center for Neurological Diseases, Beijing Tiantan Hospital, Capital Medical University, Beijing, 100070, China.

**Keywords:** Parkinson’s disease, subtypes, translation, heterogenous

## Abstract

Heterogenous clinical presentations of Parkinson’s disease have aroused several attempts in its subtyping for the purpose of strategic implementation of treatment in order to maximise therapeutic effects. Apart from *a priori* classifications based purely on motor features, cluster analysis studies have achieved little success in receiving widespread adoption. *A priori* classifications demonstrate that their chosen factors, whether it be age or certain motor symptoms, do have an influence on subtypes. However, the cluster analysis approach is able to integrate these factors and other clinical features to produce subtypes. Differences in inclusion criteria from datasets, in variable selection and in methodology between cluster analysis studies have made it difficult to compare the subtypes. This has impeded such subtypes from clinical applications. This review analysed existing subtypes of Parkinson’s disease, and suggested that future research should aim to discover subtypes that are robustly replicable across multiple datasets rather than focussing on one dataset at a time. Hopefully, through clinical applicable subtyping of Parkinson’s disease would lead to translation of these subtypes into research and clinical use.

Parkinson’s disease (PD) is a complex neurodegenerative disease that remains without a disease-modifying treatment and in most cases, without a known aetiology as well [1]. It affects more than six million people around the world [2]. Traditionally characterised by bradykinesia, resting tremor and rigidity [3], PD has been recognised in recent years as presenting with a broad spectrum of motor and non-motor symptoms [4], and varying disease progression [5]. This has led to speculation of the potential existence of distinct biologic PD subtypes. Successful PD subtyping will have important practical implications for clinicians and researchers. Subtyping patients at baseline would allow clinicians to more accurately predict prognosis, plan treatment and counsel patients. Identification of distinct subtypes would allow for more focused research into disease aetiology, pathophysiology and developing curative treatment. Unfortunately, despite numerous subtyping studies that has approached PD subtyping in various ways, efforts at subtyping has yielded little results in the field.

There have been other reviews on PD subtyping that have largely examined different perspectives of PD for possible variables that could be included in data-driven subtyping as well as methods for subtype validation [6, 7]. One recent review focused on the rise of importance of non-motor symptoms in PD subtyping, cognitive PD phenotypes and the implications for subtyping in PD that has been traditionally based on motor symptoms [6]. Another recent review examined subtyping in relation to the disease progression of PD highlighting the distinct nature of PD subtypes as opposed to subtypes being just various stages of disease progression [7]. This review will give an overview of the subtyping solutions that have been offered so far in order to comprehensively explore the challenges that face subtyping PD from being fully accepted and advocate for a future direction that looks to find consensus across subtyping studies in order to discover subtypes that are truly replicable.

The following databases were searched: Ovid MEDLINE (1946 to December 2018), Web of Science Core Collection (1900 to December 2018) and EMBASE (1947 to December 2018). The search consisted of the Medical Sub Heading term “Parkinson’s disease” used in conjunction with the keyword “subtype” or “heterogeneity”. The reference lists of articles found were also searched. The results were limited to articles in the English.

**Figure 1.**
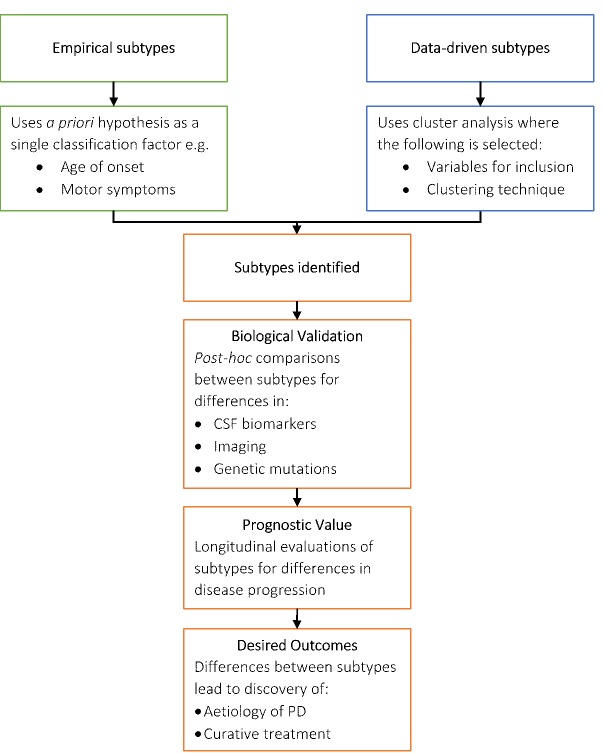
Summary of PD Subtyping. The flowchart demonstrated although the two subtyping approaches had different starting points, a common pathway of biological validation, prognosis evaluation, and the destinations (desired outcomes) was followed.

## Overview of PD Subtypes

Early proposed classifications used *a priori* hypotheses to define PD subtypes. Age of onset and motor symptoms were commonly used as the sole classification factor. As understanding of PD advanced, non-motor symptoms have been increasingly recognised as classification factors. In recent years, data driven approaches to subtyping PD have disregarded *a priori* judgements about the importance of certain variables and used statistical methods to determine subtypes. Interestingly, the outcomes of either process are similar as seen in Figure 1. Once subtypes are identified, biological evidence of differences between subtypes are sought and when found, links to possible aetiology are explored.

Subtype differences in CSF biomarkers and imaging are well documented, but subtype differences in genetic factors have been limited [8, 9]. Genetic factors contributing to PD susceptibility, disease progression and clinical severity are continually being discovered [10, 11]. This includes common variants with small effect size and risk factors, like mutations in the *GBA1* gene [12], which may influence subtype. Some genetic PD risk factors have already been associated with a higher likelihood of certain phenotypic features. Patients with *LRRK2* mutations are inclined to have tremor, responsive to levodopa and less likely to have cognitive impairment and hyposmia [13, 14]. Patients with the *Parkin* mutation are more likely to present with dystonia and hyperreflexia [15]. However, one study could not find an association between polymorphisms in *LRRK2* and *GBA1* genes and the four subtypes generated from a cluster analysis of the same patients [16]. Associations of any specific genetic factors with clinically defined subtypes remain to be identified and will be a strong priority for future subtyping studies. Although subtypes need to be valid and feasible for clinical use, subtyping can help gain insight into PD aetiology, which can assist with developing disease-modifying treatments.

## Subtypes Based on Age of Onset

Classifying PD patients using age of onset was an appealing subtyping solution due to its simplicity and its readiness to be used in a clinical setting. Young-onset PD (YOPD) refers to those who were diagnosed between ages of 21 and 40. Studies showed that this subset of the PD population tended to have slower disease progression than later onset PD [17, 18]. They often had less cognitive deterioration in comparison to those with later onset, until they reached a much older age. A study found that the advanced stages of PD, characterised by increasingly rapid decline in motor and cognitive domains, may be similar for both subtypes and concluded that the age of onset only primarily influenced the progression rate in the early stages of the disease [19]. Despite this, YOPD patients are more likely to experience earlier motor difficulties, such as potentially disabling dyskinesias, often painful dystonia, and possibly severe motor fluctuations [20, 21].

Juvenile parkinsonism describes those with clinical features of PD manifesting before 21 years of age. Juvenile parkinsonism is characterised as being commonly familial, having atypical clinical features and varied pathological findings at autopsy [22]. Whilst most later onset PD cases are idiopathic, one study found that the *parkin* gene was implicated in 77% (10 out of 13) of juvenile parkinsonism cases compared to 3% of PD patients aged over 30 years [15].

Despite established differences between juvenile parkinsonism, YOPD and later onset PD, this subtyping solution is not effective as patients with age of onset of 50 years or less represent only 5-10% of the PD population [23]. The later onset subtype remains highly heterogenous in presentation and outcomes. Furthermore, the various cut-offs of 50, 55 and 60 have been used as well to partition YOPD from later onset PD in many studies, highlighting the arbitrary nature of this subtyping method [24].

## Subtypes Based on Predominant Motor Symptoms

Similar to the age of onset subtyping system, motor subtypes use arbitrary cut-offs. Zetusky, et al. [25] first recognised the possibility of at least two motor subgroups: one with tremor as the main feature and the other with postural instability and gait difficulty (PIGD). Jankovic, et al. [26] later on defined these two motor subtypes based on the calculation of a tremor score as a mean of a set of Unified Parkinson’s Disease Rating Scale (UPDRS) items. A PIGD score was similarly calculated as a mean of another set of UPDRS items. The tremor subtype was then defined as patients with a ratio of mean tremor score/mean PIGD score greater than or equal to 1.5 and the PIGD subtype included all patients with a ratio of less than or equal to 1.0 [26]. Later studies used the same principle but based the scores on different sets of UPDRS scores and included a third subtype that did not fit within either ratio cut-offs. Sometimes the non-tremor subtype was referred to as akinetic-rigid instead of PIGD [27, 28]. This could be perhaps traced to an early study that introduced an akineto-rigid type, tremor dominant type and an equivalent type that had both features [29].

Motor subtypes have been a popular concept due to a rich body of evidence supporting an association between these subtypes with other clinically relevant features, treatment response and other areas, lending additional credibility to these subtypes [30-32]. The tremor-dominant subtype seems to present with a more benign course of disease and a slower rate of progression [33]. The akinetic-rigid subtype is linked to faster cognitive deterioration [34, 35] particularly in working memory [36]. It also has a higher risk for depression and apathy [37], hyposmia [38], prevalence of most non-motor symptoms [39], and generally a faster progression [26, 40]. Patients with the akinetic-rigid subtype have more prominent frontal lobe grey matter atrophy [41]. Tremor-dominant patients have been shown to have decreased cerebellar grey matter volume [42]. Different CSF levels of glycine, 5-hydroxyindoleacetic acid as well as Aβ42 and phosphor-tau_181_ between subtypes have been found but have yet to be confirmed in independent cohorts [27, 43].

Despite these positive findings, the approach of defining subtypes based on the ratio of two UPDRS scores is ultimately arbitrary, and results in substantial ambiguity regarding the classification of patients. Different published definitions of motor subtypes were applied to a large cohort of PD patients, which exposed considerable differences in the frequencies of the subtypes between the algorithms [4]. Additionally, the motor subtyping system has been shown to be unstable with 39% of akinetic-rigid patients and 18% of tremor-dominant patients belonging to different subtypes after one-year follow-up [44]. Due to the single classification nature of the motor subtyping system, non-motor manifestations of PD are ignored, and their inclusion could mean subtype stability.

**Table 1:** Comparison of sample characteristics of recent PD subtyping studies using cluster analysis.

Cohort clinical characteristics	Liu 2011	Van Rooden 2011	Fereshtehnejad 2015	Erro 2016	Fereshtehnejad 2017	Mu 2017
Number of patients	138	802	113	398	421	904
Inclusion criteria, in addition to PD	H&Y 1-3	None	Idiopathic PD deemed as most likely cause	*de novo*	*de novo*, H&Y 1-2, age ≥ 30,	Mixed cohort of drug-naïve and treated PD
Age (years) (means±SD)	57.47 ± 10.58	60.8-66.2 (11.0-11.3)	66.7 ± 8.9	*NS*	61.1 ± 9.7	64.28 ± 9.86
Disease duration, years (means±SD)	3 (median) range: 0.5-35.0	9.1-12.3	5.7±4.2	*NS*	6.5 ± 6.5	8.01 ± 5.60
H&Y stage	*NS*	2-3	2.5 ± 0.9	*NS*	*NS*	*NS*

H&Y: Hoehn and Yahr, SD: standard deviation, NS: Not Specified.

## Subtypes Using Data-Driven Analysis

Cluster analysis has been a popular method for subtyping PD as it can integrate a wide range of PD features, including non-motor symptoms. It is a hypothesis-free approach that approximates factor importance without bias. However, cluster analysis still relies on certain choices: variable selection, number of clusters and clustering technique. Differences in design, sample characteristics, and variables included in the analysis, seen in Table 1, Table 2, and in a review paper [7], make it difficult to compare the results of cluster analysis studies and explain the discrepancies. Erro, et al. [45] performed a cluster analysis on the Parkinson’s Progression Markers Initiative (PPMI) cohort and produced results vastly different from Fereshtehnejad, et al. [46] who did a cluster analysis on the same cohort a year later. Fereshtehnejad and Postuma [7] noted that the membership agreement rate between the two different clustering solutions was only 56%. It seems that results of such studies are highly sensitive to the study design [7].

The precision and reliability of a classification system is dependent on the quality of the input data it is based on. The data pre-processing stage maximises the quality of this data. There are several issues to consider before processing the data. 1) Missing imputation: As subjects are assessed at baseline, missing data is likely to occur in subsequent occasions when they do not attend assessments. Closest match is the best method for missing data imputation in this scenario [47]. This is when the missing value of a case at a certain timepoint is substituted with a value from other case which had the closest scores on the same variable at other timepoints. 2) Z score transformation: Variables are also generally use different units of measurement. These continuous variables are typically transformed into Z-scores to account for these differences. 3) Outliners: Identification of outliers is crucial to enhance the quality of outcomes. Some techniques used include Z-Score Method and Modified Z-Score Method where a Z-score > 2.5 - 3.0 is considered an outlier and the difference between the two methods is that the latter is more suitable for small datasets [48, 49]. The Box Plot using the Inter Quartile Range is also another possible technique for detecting outliers and preventing them for influencing results [49, 50].

**Table 2:** Comparison of methodology of recent PD subtyping studies using cluster analysis.

Methodological steps	Liu 2011	Van Rooden 2011	Fereshtehnejad 2015	Erro 2016	Fereshtehnejad 2017	Mu 2017
Data pre-processing	Standardized scores	*z* scores	*z* scores	*NS*	Normative values	Standardised scores
Clustering algorithm	K-means	Model-based	2-step	K-means	Hierarchical	K-means & Hierarchical
Basis of the determination of the number of clusters	*NS*	*NS*	Bayesian information criterion	Calinski-Harabasz pseudo-F value	Estimate, Hartigan’s rule	Various e.g. Gap Statistic and the 1-standard-error method
Cluster validation on independent sample	No	Yes	No	No	No	No
Evaluation of discriminative variables	No	Discriminant analysis	No	No	Principal component analysis	No
Follow up period, years± mean	*N/A*	*N/A*	4.5	*N/A*	2.73 ± 0.78	No
Post hoc analysis of variables not included in the cluster analysis	Yes, motor phenotype consistency	No	Yes, disease progression	Yes, ^123^[I]-FP-CIT binding values	Yes, CSF and imaging biomarkers, and disease progression	No

Once cluster profiles were identified, most studies sought out biological associations to validate them. One study used imaging and CSF biomarkers to validate their subtypes [46]. Differences between subtypes were found in MRI morphometry data that showed varying levels of atrophy in a PD-specific brain network depending on the subtype. Differences were also found in the CSF Aβ levels and Aβ:tau ratio. However, the study could not find evidence associating genetic heterogeneity with clinical presentation, conflicting with the well-documented association between *GBA1* mutations and faster motor disease progression [51]. The discrepancy could be attributed to the genetic risk score that was included in the cluster analysis [46]. It was derived from 28 variants associated with disease risk, possibly diluting the influence of variations at any loci [3]. Additionally, genetic variants that affect clinical presentation may be different from those that cause disease risk [3].

Incomplete penetrance of genetic mutations suggests that there may be an interaction between genetic variations and environmental factors in causing PD. It is also possible that environmental factors may independently influence PD neurodegeneration. In the future, studies could see if their subtypes have any environmental associations. There is evidence of an association between tobacco use and caffeine consumption with PD but neither have sufficient evidence to be considered causal. Individuals who smoked cigarettes have been found to have a reduced risk of PD [52]. Similarly, caffeine consumption decreases the risk of PD [52]. Associations with personal medical history could also be studied. Research showed that appendectomy and vagotomy are associated with reduced risk of the onset of PD [53, 54]. Whilst biological associations suggest different environmental (external) factors, along with genetic (internal) factors, might be underlying pathophysiological mechanisms for clinical subtypes of PD.

Despite numerous subtyping studies using cluster analysis, their results have not been widely used by clinicians or researchers. A systematic review of PD subtyping studies using cluster analysis from 1999 to 2008 revealed a large variability in methodologies in those studies and Table 2 shows that this pattern has continued on [55].

Another major challenge for subtyping studies using cluster analysis is that cluster analyses inherently describe variability at a group level. In each group produced, there will individuals be who do not have all of the cluster-defining features. This means that the groups generated by cluster analysis cannot be subtypes to be used in a clinical setting. Only one study attempted to address this issue by stratifying their cluster analysis findings into criteria that could subtype all the patients in the cohort [46]. Using the clinical features that defined the clusters, a categorical definition was created to assign patients to one of three subtypes. This approach would allow cluster-analysis-derived subtypes to be implemented for practical uses in personalised medicine and patient recruitment for clinical trials.

The use of cluster analysis in PD subtyping still holds potential. The shift towards standardising the application of cluster analysis to allow for comparison and developing methods for stratifying clusters for clinical use could ultimately determine subtypes useful for clinical and research purposes.

## Prognostic Evaluation of Subtypes

There are only a few studies where PD subtypes have been longitudinally evaluated and the major primary outcome used to investigate disease progression in PD have differed between studies. de Lau, et al. [56] used mortality rate to show differences in prognosis between their cluster-analysis-defined subtypes determined in the Profiling Parkinson’s disease (PROPARK) cohort. Patients of one subtype were 8 times more likely to die than those of another subtype. Predictably, the subtype that was most severely affected in all domains had the worst survival rate whilst the subtype that was only characterised by mild motor and cognitive impairments had the best survival. These differences were not accounted for by different disease durations between subtypes, however, only 37 patients (10.8%) died during the 5-year follow-up. It seems too early to conclude strongly that the survival differences found support the prognostic value of these subtypes.

To avoid the long follow-up period associated with the obvious difficulty of solely using mortality rates, Fereshtehnejad, et al. [46] created a global composite outcome score (GCO) as a single numeric indicator of prognosis similar to their previous single-centre study on PD clustering [57]. The GCO equally weighted non-motor symptoms (Movement Disorder Society, MDS-UPDRS-I), motor symptoms (MDS-UPDRS-II), motor signs (MDS-UPDRS-III), overall activities of daily living (SCWAB and England Activities of Daily Living) and global cognition (Montreal Cognitive Assessment, MoCA). The study had a much shorter follow-up duration (average of 2.7 years) but found a similar high motor and non-motor subgroup, which had fast global progression and cognitive decline. Additionally, on biomarker analysis, this group had the most rapid decline in dopamine transporter tracer uptake on single-photon emission CT (DaT-SPECT), suggesting progression of the neurodegenerative disease.

Using a different approach, Vavougios, et al. [58] set out to phenotype PD motor progression and to detect possible progression biomarkers. Motor progression was defined as a difference of at least one point in the Hoehn & Yahr (H&Y) scale between the baseline, 12 months and 36 months milestones of the PPMI study. H&Y progression events recorded at each milestone were used as cluster analysis variables. Initial CSF α-synuclein was determined to be a statistically significant cross-cluster characteristic as well as a statistically significant predictor of cluster differentiation alongside serum IGF-1 and DaT-SPECT-derived Striatal Binding Ratios. These possible biological markers of motor progression warrant further investigation in order to evaluate their possible incorporation into the prognosis of subtypes in clinical practice. By extension, there needs to be further studies to investigate the best universal outcome variable for longitudinal studies in the context of PD.

## Limitations

Datasets that are used in data driven subtyping studies have different inclusion criteria. For example, some studies only included drug naïve patients [46] whilst other studies had a mixture of drug-naïve and treated PD patients [59]. Similarly, varying information collected from the cohort can limit the variables that can be included in the clustering. This could make replication studies difficult because a required variable may not be included in the database. Ideally, a cohort should be recruited with inclusion criteria and data collection methods chosen for the purposes of finding subtypes. However, lack of resources means that recruiting and collecting data from a large sample size over a reasonable time is often not feasible. A recent study was unable to reproduce any of data-driven PD subtypes from eight published studies where their variables were matched to those available in a well-characterised cohort of patients [60].

Gender is rarely considered in subtyping although it is well-known that men have a higher incidence rate of PD than women. The ratio between males and females for age-adjusted incidence rates have been found to be from 1 to 2, with a median sitting at 1.5 [61]. However, gender differences in motor and non-motor symptoms are not apparent, except for increased incidence of depression in women in one study [62]. It is uncertain how a female-specific depressive profile will impact diagnosis and treatment [63].

## Future Direction

Most studies have used hierarchical clustering and k-means partitional methods as they are the classical clustering methods. There are other possible clustering methods that have not been applied to the field such as affinity propagation, spectral clustering density-based spatial clustering of applications with noise (DBScan), and so on. DBScan was used in a study with a voice signal dataset to classify healthy and PD patients for the purposes of diagnosis [64]. Exploration of other clustering methods may hold great potential in finding useful and replicable subtypes. The merit of these methods over other clustering methods should be determined on their replicability and whether they can predict prognosis more accurately. Machine learning techniques have also been used in the field with large datasets to predict PD progression [65, 66]. Nonetheless, the type of subtyping method chosen has not yet changed the obstacle to mainstream acceptance, which is being able to reproduce the subtypes in other datasets reliably.

The focus of this field has generally been on creating the best cluster base partition that is able to indicate appropriate treatment and predict prognosis. Shifting efforts towards comparing the numerous PD clustering studies could result in discovering reproducible subtypes or at the very least illuminate improvements to clustering methodology. Such a step has been taken in the field of breast and ovarian cancer where one study developed a framework that found replicable patient subtypes by finding consensus across cluster analysis results from multiple studies [67]. Adjusted rand index normalized mutual information, normalized variation of information and consensus clustering are potential tools to assist such comparisons.

Barriers to comparing cluster analysis studies include datasets they are based on having differing inclusion criteria and varying information collected from the cohort. With the goal of subtyping patients at diagnosis in mind, future studies should prioritise datasets that only include drug naïve patients. As PD is currently defined in clinical terms, we propose that subtyping of PD shall be based on clinical features only as a starting point. Once clinical subtypes established, the next step is to study which biomarkers or environmental factors are related to subtypes, but not use biomarkers or environmental factors as variables for clinical subtyping. In addition, cluster analysis is a highly sensitive statistical method in which additional variables in the model, rather than just the essential variables, would result in a shaky model with low statistical validity and low reproducibility. Therein, subtyping shall be based on the overall features of PD, which is reflected holistically by MDS-UPDRS - I, II, III at drug-naive state. Total scores of MDS-UPDRS-I, II, III shall be considered as initial clustering variables. Comparing this study with others could elucidate the variables that are crucial to subtyping PD. Finding a subtyping system that is universally agreed upon will allow subtypes to advance research in aetiology and disease-modifying treatment.

### Conclusion

Despite a significant body of literature on PD subtypes, there have been hardly any changes in clinical and research practice resulting from subtypes. Lack of advances in understanding aetiology and developing curative treatment have resulted in criticisms of subtyping methodology, which to date has been largely descriptive of subtypes rather than predictive of subtype or prognosis. With an increasing number of PD-specific datasets, the future of PD subtyping is finding consensus across clustering from multiple datasets to discover truly replicable subtypes and facilitate their widespread adoption into research and clinical practice.
